# Socioeconomic Status and Psychological Well-Being: Revisiting the Role of Subjective Socioeconomic Status

**DOI:** 10.3389/fpsyg.2020.01303

**Published:** 2020-06-10

**Authors:** Ginés Navarro-Carrillo, María Alonso-Ferres, Miguel Moya, Inmaculada Valor-Segura

**Affiliations:** ^1^Department of Psychology, University of Jaén, Jaén, Spain; ^2^Department of Social Psychology, Faculty of Psychology, Mind, Brain and Behavior Research Center, University of Granada, Granada, Spain

**Keywords:** subjective socioeconomic status, objective socioeconomic status, socioeconomic status, psychological well-being, social class

## Abstract

Socioeconomic status (SES) is a complex and multidimensional construct, encompassing both independent objective characteristics (e.g., income or education) and subjective people’s ratings of their placement in the socioeconomic spectrum. Within the growing literature on subjective SES belongingness and psychological well-being, subjective indices of SES have tended to center on the use of pictorial rank-related social ladders where individuals place themselves relative to others by simultaneously considering their income, educational level, and occupation. This approach, albeit consistent with the idea of these social ladders as summative or cognitive SES markers, might potentially constrain individuals’ conceptions of their SES. This research (*N* = 368; *M*_age_ = 39.67, *SD* = 13.40) is intended to expand prior investigations on SES and psychological well-being by revisiting the role of subjective SES. In particular, it (a) proposes an innovative adaptation of the traditional MacArthur Scale of subjective SES to income, education, and occupation, thus resulting in three separate social ladders; and (b) tests the empirical contribution of such three social ladders to psychological well-being. Overall, our findings showed that the novel education and occupation ladders (excluding the income ladder) are predictive of a significant part of the variance levels of psychological well-being that is not due to canonical objective metrics of SES (i.e., income, education, and occupation), or to the conventional MacArthur Scale of subjective SES. Although preliminary, these results underscore the need to further reconsider (subjective) SES-related conceptualization and measurement strategies to gather a more comprehensive understanding of the SES-psychological well-being link.

## Introduction

During the last decade, the psychology of socioeconomic status (SES) or social class, which is broadly characterized as a social stratification system derived from access to various resources (economic, social, etc.; Moya and Fiske, 2017), has experienced a remarkable growth (see Manstead, 2018). Such increased interest has been fundamentally driven by the onset of the Great Recession, which is connected to the broadening gap between the “haves” and the “have-nots” (Pfeffer et al., 2013). Indeed, in this socioeconomic climate, class disparities and their detrimental wide-ranging consequences across distinct domains are more visible (Moya and Fiske, 2017). Although it could be argued that almost all people’s psychological and social outcomes are largely influenced by their objective or perceived socioeconomic standing, ranging from food preferences (Baumann et al., 2019) and speech patterns (Kraus et al., 2019) to humor-related dispositions (Navarro-Carrillo et al., 2020) and identity (Easterbrook et al., 2020), empirical research has mainly focused on investigating the connections between SES and psychological well-being and health-related aspects (e.g., Howell and Howell, 2008; Curhan et al., 2014; Präg et al., 2016; Huang et al., 2017).

Cumulative empirical evidence has highlighted that long-established objective metrics of SES, such as income, educational level, and occupation, only show low to modest correlations with personal well-being indicators (Diener and Oishi, 2000; Howell and Howell, 2008). In contrast, a growing number of studies have revealed that subjective assessments of SES exhibit robust associations with well-being and health scores above and beyond objective SES (e.g., Adler et al., 2000; Kraus et al., 2013; Garza et al., 2017; Navarro-Carrillo et al., 2019). Within this area, while objective SES is commonly assessed using various indices of material wealth (e.g., income, education), subjective SES is primarily assessed using the MacArthur Scale of Subjective Social Status (MacArthur SSS scale; Adler et al., 2000), a pictorial format measure represented by a 10-rung social ladder on which people indicate their socioeconomic standing relative to others in society based on income, educational level, and occupation. Within psychological and health sciences, the development and subsequent consideration of this measure, whose theoretical underpinnings rely upon social comparison processes, have provided a substantial contribution in terms of the clarification of the complex nature of the SES–well-being connection. In particular, researchers have posited that the MacArthur SSS scale, insofar as it allows individuals to capture their own social standing in a personalized manner across the SES components, could represent a cognitive average of classical objective SES indices (i.e., a general marker of a person’s SES), thereby providing a more accurate estimation of SES (see Präg et al., 2016).

Given the multidimensional nature of SES mentioned above, the joint assessment of objective and subjective SES indices is invariably recommended (Kraus and Stephens, 2012; Rubin et al., 2014), as this approach would facilitate comparisons between the various facets of SES within the framework of their contribution to well-being. Indeed, that constitutes one of the major strands of research in the psychology of SES. Our paper, which is precisely embedded in that research sphere, is aimed at extending prior investigations on the SES–well-being link by presenting and testing an innovative approach to subjective SES evaluation. Although prior empirical findings indicate that conventional objective markers of SES (i.e., income, education, and occupation) are only moderately inter-correlated and thus represent independent (and not interchangeable) components of SES (Torssander and Erikson, 2010), the MacArthur SSS scale considers these objective facets of SES in a simultaneous and undifferentiated manner within the person’s social comparison process. This notion, albeit aligned with the view of the MacArthur SSS scale as a subjective general (and summative) SES marker, does not allow the scientific community to ascertain the specific weight that people attribute to each component of SES when subjectively estimating their socioeconomic position relative to others. Therefore, we aim to address this gap by (a) adapting the MacArthur SSS scale to income, educational level, and occupation and (b) unveiling whether any of these three novel social ladders (one for each SES indicator) are predictive of a significant proportion of the variance of well-being that is not attributable to neither objective measures of SES (i.e., income, education, and occupation) nor the traditional MacArthur SSS scale.

### Objective and Subjective Socioeconomic Status

Objective SES has been traditionally defined by access to material and social dimensions (Oakes and Rossi, 2003; Snibbe and Markus, 2005). Accordingly, this form of SES is usually operationalized by considering various objective indicators that may ultimately reflect differences in individuals’ access to material and social resources. In particular, among the multiple objective indices of SES, three distinctive aspects emerge quite clearly: income, educational level, and occupation (Kraus and Stephens, 2012; Baker, 2014; Manstead, 2018).

Income establishes the access path to desired services, material goods, and pleasant experiences, among other things (Lucas and Schimmack, 2009; Kraus and Stephens, 2012). In addition, and as a proof of the importance of income, prior research has shown that this indicator is connected with a broad array of psychological variables, such as social trust (Brandt et al., 2015), personality (Piff, 2014), and prosocial tendencies (Piff and Robinson, 2017). Like income, education is widely considered a canonical marker of objective SES. As Snibbe and Markus (2005) synthetized, educational level allows researchers to capture relevant sociocultural and psychosocial-related outcomes (e.g., behavioral patterns, lifestyle). Moreover, higher educational level has been linked to beneficial economic outcomes, such as, for instance, diminished financial hardship (American Psychological Association, 2007). Occupation, for its part, has been argued to be a further proxy for objective SES because of its tight connection to earnings and educational level (Duncan and Magnuson, 2012) and its capacity to differentially shape psychological experiences (American Psychological Association, 2007; Kraus and Stephens, 2012). Nevertheless, this indicator of objective SES is used less than income and education in psychological research.

Objective SES is frequently measured in undergraduate samples (by utilizing a global index encompassing family income and parental educational level; Kraus and Keltner, 2009; Côté et al., 2017) and community-based samples (by including a specific objective indicator or by building a composite index defined by the combination of some of these dimensions, particularly income and educational level; Kraus and Park, 2014; Navarro-Carrillo et al., 2018). Earlier research revealed that these objective facets of SES are moderately inter-correlated (Singh-Manoux et al., 2003), which suggests that these indices should be distinguishable. In this vein, the Report of the APA Task Force on Socioeconomic Status (American Psychological Association, 2007) stated that “it is generally more informative to assess the different dimensions of SES and understand how each contributes to an outcome under study rather than merge the measures” (p. 11).

SES is not exclusively shaped by material resources. Indeed, current approaches underscore that subjective assessments founded on social comparison processes (e.g., determining one’s own socioeconomic position relative to that of other individuals or groups) play a pivotal role in shaping SES (Boyce et al., 2010; Kraus, 2018). Consistent with this emerging perspective, subjective SES is conceptualized as individuals’ perceptions pertaining to their standing in the social hierarchy relative to others (Adler et al., 2000; Kraus et al., 2012).

Although different methods of assessing subjective SES exist (see Rubin et al., 2014), one of the few such tools explicitly based on a relative social comparison process is the MacArthur SSS scale (Adler et al., 2000). Furthermore, this measure is the dominant means of evaluating subjective SES (Cundiff and Matthews, 2017). Using this graphical 10-rung ladder, which represents ascending positions based on income, educational level, and occupation, individuals estimate their SES by marking the rung where they place themselves relative to others in society in general or in a specific social group or community.

As in the case of objective SES, subjective SES is commonly assessed using the MacArthur SSS scale in undergraduate (Jury et al., 2019; Loeb and Hurd, 2019) and community-based (Bjornsdottir et al., 2017; Wang et al., 2019) samples. However, studies that use this measure among adolescents are becoming more frequent (e.g., Joffer et al., 2019; Moor et al., 2019). Prior research has constantly shown that the MacArthur SSS scale exhibits mostly moderated associations with traditional objective SES indicators (Adler et al., 2000; Ostrove et al., 2000), thus providing evidence of its conceptual and empirical differentiation from objective SES.

### Objective and Subjective Socioeconomic Status and Psychological Well-Being

The analysis of the empirical connection between SES and psychological well-being and health has been the focus of much controversy across the various disciplines interested in addressing this issue. This stems at least in part from the various approaches used to conceptualize and measure SES as a relevant factor for well-being and health. Notwithstanding the above, examining the socioeconomic determinants of psychological well-being—which broadly refers to optimal human functioning and the eagerness to reach meaningful vital objectives (Ruini and Cesetti, 2019)—became of particular interest because, throughout different studies, its desirable effects on various personal domains have been substantiated. For instance, higher levels of psychological well-being have been related to positive family experiences and optimal biological functioning (Ryff, 2014), as well as with reduced depression levels (Ryff and Keyes, 1995; Ruini and Cesetti, 2019).

Many studies have amassed empirical evidence on the positive relationship between SES (as measured by objective, classical indices of material wealth) and psychological well-being/health-related factors (e.g., Diener and Oishi, 2000; Diener and Biswas-Diener, 2002; Diener et al., 2003; Vera-Villarroel et al., 2015). Nevertheless, it is important to note that the strength of such associations is relatively modest. For instance, Howell and Howell (2008), in a meta-analytic research analyzing the relationship between objective SES and personal well-being in a total of 111 independent samples from 54 countries worldwide, revealed that the average estimated association of these variables was approximately *r* = 0.13.

Studies that examined the role of subjective SES (as measured by the MacArthur SSS scale or equivalent scales) have provided valuable comprehensive knowledge on the SES–well-being/health connection. Classical empirical works, such as those developed by Adler et al. (2000), Goodman et al. (2003), Singh-Manoux et al. (2003), or Cohen et al. (2008), established the foundations on which more recent investigations were built. This groundbreaking research has shown that subjective SES is, compared to objective SES, a stronger predictor of psychological functioning indicators (e.g., control over life) and physiological outcomes (e.g., heart rate and sleep latency) among healthy white women (Adler et al., 2000), body mass index among adolescents (Goodman et al., 2003), ill health among civil service employees (Singh-Manoux et al., 2003), and susceptibility to upper respiratory infection among healthy men and women (Cohen et al., 2008). Importantly, these effects of subjective SES on well-being and health-related outcomes were independent of the respondents’ objective SES, thereby by providing solid preliminary support for the independent contribution of subjective socioeconomic standing to well-being/health. Results from subsequent investigations are also in keeping with those mentioned above. Thus, the stronger connection of subjective SES, as compared to conventional objective markers of SES, with various psychological well-being and health-related aspects has recently been proved by valuable research findings. For instance, subjective SES (as measured by a social ladder comparable to the MacArthur SSS scale) was found to be associated with psychological well-being and self-perceived health even after controlling for objective SES across 29 countries (Präg et al., 2016). Along the same lines, Cundiff and Matthews (2017), after analyzing a total of 31 studies, demonstrated that subjective SES (assessed by the MacArthur SSS scale) had a unique relationship with physical health in adults over and above canonical objective indices of SES. Similarly, Tan et al. (in press) examined the link between objective and subjective SES and well-being in a total of 336 independent samples. Their results not only confirmed that the estimated subjective SES–well-being association was significantly larger (almost twice) than that of the objective SES–well-being association, but also illuminated differences that depended on the type of objective and subjective SES measure. In particular, in terms of objective SES, their data showed that the meta-analytic effect size corresponding to the relationship of income with well-being was higher than that of education. Regarding subjective SES measures, the meta-analytic effect size of the relation between the MacArthur SSS scale and well-being was higher than that of the connection between the perceived SES category and well-being, thus verifying the crucial role of the MacArthur SSS scale.

In summary, there is increasingly solid evidence that, beyond the objective material substance of SES, individuals’ subjective perceptions of their position in the socioeconomic hierarchy capture specific differences in well-being/health. However, to the best of our knowledge, no empirical investigation has yet addressed whether the subjective placement within three distinctive graphical social ladders based on income, education, and occupation, rather than within a unique social ladder that considers these three (empirically distinguishing) dimensions of SES together, would uniquely account for psychological well-being scores. Adding to the growing literature on the determination of the ability of various SES indicators to predict well-being, we surmise that these new exploratory approaches could refine subjective SES measurement by elucidating the particular role of such differentiated SES components. Thus, such an approach would facilitate the gathering of comprehensible information pertaining to the need (or absence thereof) for further research to evaluate subjective SES by considering income, education, and occupation using separate social ladders.

## Materials and Methods

### Participants

The sample was composed of 368 participants (*M*_age_ = 39.67, *SD* = 13.40, range from 18 to 90), of whom 64.4% were women, 34.5% were men, and 1.1% did not identify themselves as women or men. A total of 19.8% of the participants were single, 17.9% were dating, 11.4% were cohabiting, 42.9% were married, 5.4% were divorced, and 2.4% were widowers. A sensitive power analysis was conducted using linear multiple regressions: fixed model, R^2^ deviation from zero in G^∗^Power (Faul et al., 2009) to determine our ability to detect the contribution of each SES indicator on psychological well-being. Taking our sample (*N* = 368, α = 0.05) into account, the sensitivity analysis suggests that effect sizes of *f*^2^ ≥ 0.04 are necessary to produce power at the 0.80 level.

### Procedure

A snowball sampling procedure via online administration was used to recruit the participants. Specifically, before the questionnaire was distributed, undergraduate students at a university in southeastern Spain were trained in sampling methods. Collaborators were asked to distribute the questionnaire only to adults of legal age (≥ 18 years). Afterward, they contacted potential respondents (e.g., acquaintances) and provided them with a brief description of the study. Once the participants agreed to participate in the study, they were given access to the online survey. At the beginning of the survey, the participants received information that emphasized the principles of confidentiality and anonymity in this research, their voluntary participation, and the estimated duration. In addition, they were given the e-mail address of one of the researchers in the case they needed to resolve any issues arising from their participation. After signing an informed consent form, the participants completed the questionnaire. Finally, the undergraduate students in charge of distributing the online survey among potential respondents received partial academic credit in exchange for their participation. The study was approved by the ethical committee of the southeastern Spanish university and carried out in compliance with the ethical standards of the Declaration of Helsinki.

### Measures

#### Psychological Well-Being

The Spanish adaptation of Ryff’s Psychological Well-Being Scales (PWBS; Díaz et al., 2006) was used. It consists of 29 items rated on a 6-point Likert scale ranging from 1 (*strongly disagree*) to 6 (*strongly agree*) that covered six subscales: self-acceptance (e.g., “When I review the story of my life I am happy with how things have turned out”; α = 0.80), positive relationships with others (e.g., “I feel that my friends bring me many things”; α = 0.68), autonomy (e.g., “I have confidence in my opinions even if they are contrary to the general consensus”; α = 0.62), environmental mastery (e.g., “In general, I feel that I am responsible for the situation in which I live”; α = 0.63), purpose in life (e.g., “I have clear the direction and purpose of my life”; α = 0.81), and personal growth (e.g., “I have the feeling that over time I have grown as a person”; α = 0.77). High scores indicated high levels of psychological well-being. The PWBS has six subscales grouped into a second-order factor called global psychological well-being (Ryff and Keyes, 1995). Because the proposed six-dimensional structure with a second-order general factor has been confirmed with Spanish samples (Díaz et al., 2006; Van Dierendonck et al., 2008), we also computed the items’ average as a global indicator of psychological well-being (α = 0.88).

#### Subjective SES

The traditional 10-rung social ladder *MacArthur SSS scale* (Adler et al., 2000) was administered. Participants were asked to select the rung that represented their position in the social hierarchy relative to others in society in terms of income, educational level, and occupation. High numbers were indicative of higher placement on this social ladder.

##### MacArthur SSS scale adaptations to income, education, and occupation

Based on the traditional MacArthur SSS scale, we created three pictorial social-related ladders to independently tap people’s subjective perceptions of their (a) income, (b) educational level, and (c) occupational status. Thus, respondents were presented three adapted 10-rung social ladders, one for each SES indicator: (a) *Income ladder*. This ladder assessed the individuals’ subjective perceptions of their position in the social hierarchy relative to others in society in terms of income-money. Participants were asked to indicate the rung of this ladder on which they believed they stood, considering that individuals at the top of the ladder would have the most income-money, whereas those at the bottom would have the least income-money; (b) *Education ladder.* This ladder evaluated the individuals’ subjective perceptions of their position in the social hierarchy relative to others in society terms of educational level. Participants were asked to select the rung of this ladder on which they perceived they stood, taking into account that people at the top of the ladder would have the most education, whereas those at the bottom would have the least education; (c) *Occupation ladder*. This ladder assessed the individuals’ subjective perceptions of their position in the social hierarchy relative to others in society in terms of occupational status. Participants had to select the rung of this ladder on which they perceived they stood, considering that individuals at the top of the ladder would have the best jobs, whereas those at the bottom would have the worst jobs or no job.

#### Objective SES

##### Income

Respondents indicated their family’s approximate net monthly income, considering all income sources (e.g., salaries, pensions, scholarships, rental income, etc.). Income was coded into ten categories, from 1 (<650€) to 10 (>5.800€).

##### Educational level

Participants indicated the highest level of education they had completed. Educational level was coded into eight categories, from 1 (primary school) to 8 (doctoral degree).

##### Occupation

Participants indicated which professional occupation best described the type of work they do (European Social Survey, 2018). In this research, occupational status was coded into ten categories, from 1 (unemployed) to 10 (technical professional occupations).

The participants’ distribution in terms of these indices of objective SES is given in the results section.

### Statistical Analyses

First, frequency distribution analyses and reliabilities were obtained. Second, Pearson product-moment correlations were performed to test the relationships among the objective and subjective SES indicators and the various psychological well-being dimensions. Before we conducted the hierarchical regression analyses, age, and objective and subjective SES measures were standardized. Then, as an initial check, we confirmed that the collinearity statistics did not exceed the recommended values (Akinwande et al., 2015). Afterward, we performed the hierarchical regression analyses, in which we entered common sociodemographic factors (i.e., gender, age, and marital status) in Step 1 (method: enter). Then, we added objective SES indicators as predictors in Step 2 (method: enter). We included the traditional MacArthur SSS scale in Step 3 (method: enter). Lastly, we entered the new proposed ladders for income, educational level, and occupation in Step 4 (method: enter) to estimate their added value in explaining variance in the criterion variables and to determine their potential unique contribution to psychological well-being above and beyond demographics, objective SES, and the MacArthur SSS scale. We separately introduced self-acceptance, positive relationships with others, autonomy, environmental mastery, purpose in life, personal growth, and global psychological well-being as criteria throughout each regression analysis. We computed the abovementioned analyses using SPSS Version 21.

## Results

### Description of Objective and Subjective SES Indicators

Regarding the objective SES indicators, the respondents’ income distribution were as follows: <650€ (5.7%), 651€–1300€ (22.8%), 1301€–1950€ (21.7%), 1951€–2600€ (20.9%), 2601€–3250€ (9.5%), 3251€–3900€ (8.4%), 3901€–4550€ (6%), 4551€–5200€ (2.2%), 5201€–5800€ (1.4%), and >5.800 € (1.4%). For educational level, we found the following distribution: primary school (9%), secondary education (5.7%), vocational training (14.4%), bachelor’s degree (9.5%), incomplete university degree (19.6%), university degree (27.4%), master’s degree (10.3%), and doctoral degree (4.1%). Finally, concerning participants’ occupation, we found the following: unemployed (17.4%), agricultural work (e.g., agricultural or livestock worker, day laborer, tractor driver, fisherman, etc.; 3.5%), unskilled worker (e.g., pawn, loading or unloading waiter, unskilled factory worker, etc.; 2.2%), semi-skilled worker (e.g., bricklayer, bus driver, cannery operator, carpenter, metallurgy worker, baker, etc.; 3.5%), skilled worker (e.g., foreman, mechanic, copyist, turner or milling machine, electrician, etc.; 4.6%), occupations related to the service sector (e.g., restaurant owner, police officer, waiter, caretaker, hairdresser, armed forces, etc.; 15.2%), commercials (e.g., sales manager, store owner, store clerk, insurance agent, etc.; 8.2%), office work (e.g., secretary, administrative, accounting, etc.; 6%), senior management administrative occupations (e.g., banking executive, executive of a large company or organization, senior public administration officer, delegate, union, etc.; 3.8%), and technical professional occupations (e.g., doctor, teacher, engineer, artist, financial director, etc.; 33.7%).

**FIGURE 1:**
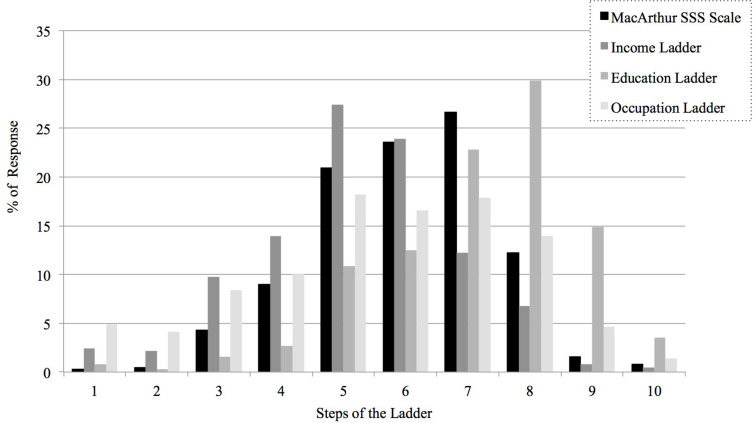
Frequency distribution of each subjective SES indicator.

The frequency distribution of the subjective SES indicators is represented graphically in Figure 1. As this figure illustrates, some differences in the distribution rate of the traditional MacArthur SSS scale compared to each novel ladder for income, education, and occupation, as well as between these new ladders, can be observed. For instance, the mean of the traditional MacArthur SSS scale was 6.04 (*SD* = 1.47); the means of the income, education, and occupation ladders were 5.25 (*SD* = 1.63), 7.15 (*SD* = 1.62), and 5.61 (*SD* = 2.11), respectively. Responses ranged from 1 to 10 for all ladders.

### Associations Between Objective and Subjective SES Indicators

Pearson correlations among objective and subjective SES indicators are given in Table 1. Objective SES indices (i.e., income, education, and occupation) were positively inter-correlated with each other (*rs* ≥ 0.25, *p* < 0.001). Furthermore, the traditional MacArthur SSS scale was found to be significantly and positively correlated with all the objective SES indicators: *r*_income_ = 0.30, *p* < 0.001; *r*_education_ = 0.12, *p* = 0.022; and *r*_occupation_ = 0.24, *p* < 0.001. In addition, the traditional MacArthur SSS scale was also positively related to the novel income, education, and occupation ladders. Specifically, in this case, the correlations of the traditional MacArthur SSS scale with the income, education, and occupation ladders were higher: *r*_income ladder_ = 0.62, *p* < 0.001; *r*_education ladder_ = 0.36, *p* < 0.001; and *r*_occupation ladder_ = 0.55, *p* < 0.001. Nonetheless, the coefficients were lower than 0.70, ruling out multicollinearity concerns. This pattern of correlations seems to indicate that although the traditional MacArthur SSS scale and the new proposed ladders for income, educational level, and occupation undoubtedly share components, they also differ. This supports the existence of differences between such subjective SES measures. Finally, the income, education, and occupation ladders exhibited significant and positive weak-to-moderate relationships with objective SES factors (i.e., income, education, and occupation). Among these, the only exception was the connection between educational level and the income ladder (*ps* > 0.05).

**TABLE 1:** Pearson correlations between objective and subjective SES indicators.

	**1**	**2**	**3**	**4**	**5**	**6**	**7**
***Objective SES measures***							
1. Income	–						
2. Education	0.25***	–					
3. Occupation	0.37***	0.38***	–				
*Subjective SES measures*							
4. MacArthur SSS scale	0.30***	0.12*	0.24***	–			
5. Income ladder	0.32***	0.03	0.24***	0.62***	–		
6. Education ladder	0.23***	0.51***	0.26***	0.36***	0.32***	–	
7. Occupation ladder	0.35***	0.12*	0.42***	0.55***	0.55***	0.28***	–
*Mdn*	3.00	5.00	7.00	6.00	5.00	7.00	6.00

*N* = 368. SES, Socioeconomic Status; SSS, Subjective Socioeconomic Status. *p < 0.05, **p < 0.01, ***p < 0.001.

### Associations Between Objective and Subjective SES Indicators and Psychological Well-Being

Pearson correlations among objective and subjective SES indicators and psychological well-being scales are given in Table 2. The traditional MacArthur SSS scale did not correlate with autonomy (*r* = 0.02, *p* = 0.712). However, this measure of subjective SES was positively related to self-acceptance, positive relationships with others, environmental mastery, purpose in life, personal growth, and global psychological well-being. Specially, weak correlations coefficients were found for the link between the traditional MacArthur SSS scale and positive relationships with others (*r* = 0.15, *p* = 0.003) and personal growth (*r* = 0.16, *p* = 0.003). More intense associations emerged between the traditional MacArthur SSS scale and self-acceptance, environmental mastery, purpose in life, and global psychological well-being (*rs* ≥ 0.25, *p* < 0.001).

**TABLE 2:** Bivariate correlations of objective and subjective SES indicators with psychological well-being.

	**Self-acceptance**	**Positive relationships**	**Autonomy**	**Environmental mastery**	**Purpose in life**	**Personal growth**	**Global psychological well-being**
*Objective SES measures*							
1. Income	0.20***	0.14**	0.12*	0.20***	0.14**	0.04	0.20***
2. Education	0.07	0.23***	0.14**	0.10	0.10	0.18**	0.19***
3. Occupation	0.16**	0.07	0.03	0.16**	0.18**	0.08	0.16**
*Subjective SES measures*							
4. MacArthur SSS scale	0.35***	0.15**	0.02	0.25***	0.25***	0.16**	0.26***
5. Income ladder	0.29***	0.12*	0.02	0.29***	0.24***	0.07	0.23***
6. Education ladder	0.21***	0.26***	0.19***	0.20**	0.21***	0.25***	0.30***
7. Occupation ladder	0.33***	0.13*	0.01	0.33***	0.29***	0.17**	0.28***

*N* = 368. SES, Socioeconomic Status; SSS, Subjective Socioeconomic Status. *p < 0.05, **p < 0.01, ***p < 0.001.

Focusing on the novel income, education, and occupation ladders, our results showed that the income ladder did not correlate with autonomy (*r* = 0.02, *p* = 0.726) or personal growth (*r* = 0.07, *p* = 0.175). However, it was positively and significantly associated with self-acceptance, positive relationships with others, environmental mastery, purpose in life, and global psychological well-being (*rs* ≥ 0.12). Note that the same pattern of correlations was found for income (*rs* ≤ 0.20) as an objective feature of SES. Nevertheless, these associations were stronger for the subjective income ladder. Likewise, the education ladder was positively and significantly related with each component of well-being (*rs* ≥ 0.19), as well as with the global psychological well-being factor (*rs* = 0.30, *p* < 0.001). By contrast, objective educational level did not correlate with self-acceptance (*r* = 0.07, *p* = 0.172), environmental mastery (*r* = 0.10, *p* = 0.065), and purpose in life (*r* = 0.10, *p* = 0.055). This indicator of objective SES was found to correlate with the rest of measures of psychological well-being (*rs* ≤ 0.23); however, these associations were stronger for the education ladder. Finally, the occupation ladder did not correlate with autonomy (*r* = 0.01, *p* = 0.822). Nevertheless, it was positively and significantly associated with self-acceptance, positive relationships with others, environmental mastery, purpose in life, personal growth, and global psychological well-being (*rs* ≥ 0.13). A different pattern of associations was found for occupation as an objective indicator of SES. Occupation did not correlate with autonomy, positive relationships with others, and personal growth (*rs* ≤ 0.08); however, it showed a positive association with self-acceptance, environmental mastery, purpose in life, and general psychological well-being (*rs* ≤ 0.18).

### Hierarchical Regression Analyses Predicting Psychological Well-Being

Table 3 through 9 give the findings from the set of multiple hierarchical regression analyses predicting each component of psychological well-being (as well as its general indicator) from demographic factors (i.e., gender, age, and marital status), and the objective and subjective SES measures as predictors.

**TABLE 3:** Hierarchical regression analysis predicting self-acceptance.

**Predictors**	***ΔR^2^***	**ß**	***t***	**CI (95%)**
**Step 1: *Demographics***	0.02			
Age		0.05	0.84	[−0.06, 0.15]
Gender		0.10	1.90	[−0.01, 0.38]
Marital status		–0.04	–0.65	[−0.32, 0.16]
**Step 2: *Objective SES measures***	0.04**			
Age		0.04	0.79	[−0.07, 0.15]
Gender		0.08	1.60	[−0.04, 0.35]
Marital status		0.00	0.08	[−0.23, 0.25]
Income		0.15**	2.49	[0.03, 0.26]
Education		–0.01	–0.23	[−0.13, 0.10]
Occupation		0.11	1.77	[−0.01, 0.23]
**Step 3: *MacArthur SSS scale***	0.08***			
Age		0.02	0.34	[−0.09, 0.12]
Gender		0.07	1.32	[−0.06, 0.31]
Marital status		0.01	0.24	[−0.20, 0.26]
Income		0.07	1.31	[−0.04, 0.19]
Education		–0.02	–0.38	[−0.13, 0.09]
Occupation		0.07	1.19	[−0.05, 0.19]
MacArthur SSS scale		0.30***	5.63	[0.20, 0.40]
**Step 4: *New social ladders***	0.02**			
Age		0.02	0.34	[−0.09, 0.12]
Gender		0.05	1.07	[−0.08, 0.28]
Marital status		0.02	0.29	[−0.20, 0.26]
Income		0.05	0.90	[−0.06, 0.17]
Education		–0.05	–0.85	[−0.18, 0.07]
Occupation		0.02	0.35	[−0.10, 0.14]
MacArthur SSS scale		0.19**	2.79	[0.06, 0.32]
Income ladder		0.02	0.31	[−0.12, 0.16]
Education ladder		0.09	1.45	[−0.03, 0.22]
Occupation ladder		0.16**	2.34	[0.03, 0.29]

*N* = 368; SES, Socioeconomic Status; SSS, Subjective Socioeconomic Status; Gender (1 = females, 2 = males); Marital Status (1 = involved in a romantic relationship, 2 = non-involved in a relationship). Standardized regression coefficients are reported. All VIFs ≤ 1.96. *p < 0.05, **p < 0.01, ***p < 0.001.

#### Self-Acceptance

When demographic characteristics were controlled in Step 1 (see Table 3), our results revealed that, among the indicators of objective SES entered in Step 2, only income exerted predictive utility (β = 0.15, *p* = 0.013; 95% CI [0.03, 0.26]) regarding participants’ self-acceptance. Thus, the higher the income, the greater the levels of self-acceptance.

Regarding the traditional MacArthur SSS scale, which was included in Step 3, a respondent’s higher placement on this ladder (β = 0.30, *p* < 0.001; 95% CI [0.20, 0.40]) was indicative of a greater level of self-acceptance.

When we focused on the income, education, and occupation ladders (see Table 3), our results showed that, after controlling for demographics, objective metrics of SES (i.e., income, education, and occupation), and the MacArthur SSS scale, the occupation ladder emerged as a significant predictor of self-acceptance (β = 0.16, *p* = 0.020; 95% CI [0.03, 0.29]). Therefore, the higher the respondent’s placement in the occupation ladder, the greater their score on self-acceptance. The addition of these new social ladders in Step 4 accounted for incremental criterion variance (2%), *F*(3,341) = 3.16, *p* = 0.025.

#### Positive Relationships

As with self-acceptance (see Table 4), income was found to predict positive relationships (β = 0.12, *p* = 0.044; 95% CI [0.00, 0.23]). However, in this case, educational level also showed predictive utility regarding positive relationships (β = 0.20, *p* = 0.001; 95% CI [0.08, 0.31]).

**TABLE 4:** Hierarchical regression analysis predicting positive relationships.

**Predictors**	***ΔR^2^***	**ß**	***t***	**CI (95%)**
**Step 1: *Demographics***	0.01			
Age		–0.09	–1.63	[−0.20, 0.02]
Gender		–0.04	–0.66	[−0.26, 0.13]
Marital status		0.05	0.86	[−0.13, 0.34]
**Step 2: *Objective SES measures***	0.06***			
Age		–0.06	–1.04	[−0.16, 0.05]
Gender		–0.05	–1.03	[−0.29, 0.09]
Marital status		0.05	0.97	[−0.12, 0.36]
Income		0.12*	2.02	[0.00, 0.23]
Education		0.20**	3.41	[0.08, 0.31]
Occupation		–0.04	–0.58	[−0.15, 0.08]
**Step 3: *MacArthur SSS scale***	0.02*			
Age		–0.07	–1.24	[−0.18, 0.04]
Gender		–0.06	–1.18	[−0.30, 0.08]
Marital status		0.06	1.04	[−0.11, 0.36]
Income		0.09	1.47	[−0.03, 0.20]
Education		0.19**	3.37	[0.08, 0.31]
Occupation		–0.05	–0.85	[−0.17, 0.07]
MacArthur SSS scale		0.13*	2.38	[0.02, 0.24]
**Step 4: *New social ladders***	0.02			
Age		–0.06	–1.03	[−0.17, 0.05]
Gender		–0.07	–1.34	[−0.32, 0.06]
Marital status		0.05	0.98	[−0.12, 0.36]
Income		0.07	1.25	[−0.04, 0.19]
Education		0.14*	2.08	[0.01, 0.27]
Occupation		–0.08	–1.22	[−0.20, 0.05]
MacArthur SSS scale		0.05	0.76	[−0.09, 0.19]
Income ladder		0.00	0.03	[−0.14, 0.14]
Education ladder		0.13*	2.01	[0.00, 0.26]
Occupation ladder		0.08	1.13	[−0.06, 0.22]

*N* = 368; SES, Socioeconomic Status; SSS, Subjective Socioeconomic Status; Gender (1 = females, 2 = males); Marital Status (1 = involved in a romantic relationship, 2 = non-involved in a relationship). Standardized regression coefficients are reported. All VIFs ≤ 1.96. *p < 0.05, **p < 0.01, ***p < 0.001.

The MacArthur SSS scale contributed to the prediction of positive relationships (β = 0.13, *p* = 0.018; 95% CI [0.02, 0.24]). Thus, participants who placed themselves higher on the MacArthur SSS scale reported increased positive relationships.

As for the novel indicators of subjective SES entered in the last step (see Table 4), only the education ladder yielded a significant contribution to the prediction of positive relationships (β = 0.13, *p* = 0.045; 95% CI [0.00, 0.26]) above and beyond demographics, objective SES indicators, and the MacArthur SSS scale. Albeit not significant, *F*(3,341) = 2.02, *p* = 0.112, the inclusion of the income, education, and occupation ladders in the regression equation accounted for a 2% variance in the criterion measure. Furthermore, as shown in Table 4, the traditional MacArthur SSS scale no longer showed predictive utility regarding positive relationships in this last step (β = 0.05, *p* = 0.447; 95% CI [−0.09, 0.19]).

#### Autonomy

As can be seen in Table 5, and as in all other such previous cases, a higher income was indicative of greater levels of autonomy (β = 0.12, *p* = 0.040; 95% CI [0.01, 0.23]), even after accounting for respondents’ demographic characteristics.

**TABLE 5:** Hierarchical regression analysis predicting autonomy.

**Predictors**	***ΔR^2^***	**ß**	***t***	**CI (95%)**
**Step 1: *Demographics***	0.03*			
Age		0.07	1.32	[−0.04, 0.18]
Gender		0.12*	2.23	[0.03, 0.41]
Marital status		0.10	1.80	[−0.02, 0.45]
**Step 2: *Objective SES measures***	0.03*			
Age		0.10	1.75	[−0.01, 0.20]
Gender		0.10*	1.96	[0.00, 0.38]
Marital status		0.11	1.94	[−0.00, 0.48]
Income		0.12*	2.06	[0.01, 0.23]
Education		0.11	1.95	[−0.00, 0.23]
Occupation		–0.06	–0.96	[−0.18, 0.06]
**Step 3: *MacArthur SSS scale***	0.00			
Age		0.10	1.79	[−0.01, 0.21]
Gender		0.11*	1.99	[0.00, 0.39]
Marital status		0.11	1.92	[−0.01, 0.48]
Income		0.13*	2.14	[0.01, 0.24]
Education		0.12	1.96	[0.00, 0.23]
Occupation		–0.05	–0.89	[−0.17, 0.07]
MacArthur SSS scale		–0.03	–0.58	[−0.14, 0.08]
**Step 4: *New social ladders***	0.03*			
Age		0.13*	2.30	[0.02, 0.24]
Gender		0.10	1.95	[−0.00, 0.38]
Marital status		0.09	1.70	[-0.03, 0.45]
Income		0.13*	2.20	[0.01, 0.25]
Education		0.01	0.18	[−0.12, 0.14]
Occupation		–0.05	–0.77	[−0.17, 0.08]
MacArthur SSS scale		–0.06	–0.82	[−0.19, 0.08]
Income ladder		–0.05	–0.71	[−0.19, 0.09]
Education ladder		0.21**	3.16	[0.08, 0.34]
Occupation ladder		–0.02	–0.29	[−0.16, 0.12]

*N* = 368; SES, Socioeconomic Status; SSS, Subjective Socioeconomic Status; Gender (1 = females, 2 = males); Marital Status (1 = involved in a romantic relationship, 2 = non-involved in a relationship). Standardized regression coefficients are reported. All VIFs ≤ 1.96. *p < 0.05, **p < 0.01, ***p < 0.001.

Turning now to the MacArthur SSS scale (see Table 5), our results did not yield a significant contribution of this indicator of subjective SES to the prediction of autonomy (β = −0.03, *p* = 0.561; 95% CI [−0.14, 0.08]). Moreover, the inclusion of the traditional MacArthur SSS scale in this third step did not significantly account for incremental variance (0%), *F*(1,344) = 0.34, *p* = 0.561.

As with positive relationships, the education ladder emerged as a predictor of the participants’ levels of autonomy even after accounting for demographics, objective SES measures, and the MacArthur SSS scale (see Table 5). In particular, respondents who placed themselves higher on this education ladder were more inclined to report greater scores on autonomy (β = 0.21, *p* = 0.002; 95% CI [0.08, 0.34]). Importantly, the addition of the new social ladders (i.e., the income, education, and occupation) in Step 4 accounted for incremental criterion variance (3%), *F*(3,341) = 3.36, *p* = 0.019.

#### Environmental Mastery

As in the preceding cases, income was found to predict environmental mastery beyond demographics (β = 0.15, *p* = 0.012; 95% CI [0.03, 0.26]). Therefore, higher income was indicative of greater scores on environmental mastery.

As can be seen in Table 6, the MacArthur SSS scale significantly contributed to the prediction of environmental mastery (β = 0.17, *p* = 0.003; 95% CI [0.06, 0.27]), even after accounting for demographics and objective SES.

**TABLE 6:** Hierarchical regression analysis predicting environmental mastery.

**Predictors**	***ΔR^2^***	**ß**	***t***	**CI (95%)**
**Step 1: *Demographics***	0.02*			
Age		0.15**	2.68	[0.04, 0.25]
Gender		0.03	0.53	[−0.14, 0.24]
Marital status		–0.02	–0.41	[−0.29, 0.19]
**Step 2: *Objective SES measures***	0.04**			
Age		0.15**	2.82	[0.05, 0.26]
Gender		0.01	0.18	[−0.17, 0.21]
Marital status		0.01	0.20	[−0.21, 0.26]
Income		0.15**	2.52	[0.03, 0.26]
Education		0.05	0.82	[−0.07, 0.16]
Occupation		0.08	1.37	[−0.04, 0.20]
**Step 3: *MacArthur SSS scale***	0.02**			
Age		0.14**	2.58	[0.03, 0.25]
Gender		0.00	–0.01	[−0.19, 0.19]
Marital status		0.02	0.29	[−0.20, 0.27]
Income		0.11	1.83	[−0.01, 0.22]
Education		0.04	0.75	[−0.07, 0.16]
Occupation		0.06	1.04	[−0.06, 0.18]
MacArthur SSS scale		0.17**	3.02	[0.06, 0.27]
**Step 4: *New social ladders***	0.05***			
Age		0.13*	2.45	[0.03, 0.24]
Gender		–0.02	–0.35	[−0.22, 0.15]
Marital status		0.02	0.45	[−0.18, 0.28]
Income		0.07	1.16	[−0.05, 0.18]
Education		0.02	0.30	[−0.11, 0.15]
Occupation		–0.01	–0.20	[−0.13, 0.11]
MacArthur SSS scale		–0.01	–0.20	[−0.15, 0.12]
Income ladder		0.09	1.22	[−0.05, 0.22]
Education ladder		0.09	1.43	[−0.03, 0.22]
Occupation ladder		0.23**	3.40	[0.10, 0.36]

*N* = 368; SES, Socioeconomic Status; SSS, Subjective Socioeconomic Status; Gender (1 = females, 2 = males); Marital Status (1 = involved in a romantic relationship, 2 = non-involved in a relationship). Standardized regression coefficients are reported. All VIFs ≤ 1.96. *p < 0.05, **p < 0.01, ***p < 0.001.

As with self-acceptance, only the occupation ladder yielded a significant contribution to the prediction of environmental mastery (β = 0.23, *p* = 0.001; 95% CI [0.10, 0.36]) beyond demographics, objective SES indicators, and the traditional MacArthur SSS scale (see Table 6). In addition, the inclusion of the income, education, and occupation ladders in this last step accounted for 5% of the variance in the criterion measure, *F*(3,341) = 6.85, *p* < 0.001. The regression coefficient of the MacArthur SSS scale was no longer significant in this last step (β = −0.01, *p* = 0.842; 95% CI [−0.15, 0.12]).

#### Purpose in Life

Unlike the cases described above, we found none of the objective indices of SES (i.e., income, education, and occupation) predicted purpose in life beyond the respondents’ demographics (see Table 7).

**TABLE 7:** Hierarchical regression analysis predicting purpose in life.

**Predictors**	***ΔR^2^***	**ß**	***t***	**CI (95%)**
**Step 1: *Demographics***	0.02			
Age		0.05	0.97	[−0.05, 0.16]
Gender		0.04	0.82	[−0.11, 0.27]
Marital status		−0.12*	–2.24	[−0.50, -0.03]
**Step 2: *Objective SES measures***	0.03*			
Age		0.05	0.97	[−0.05, 0.16]
Gender		0.03	0.61	[−0.13, 0.25]
Marital status		–0.10	–1.83	[−0.46, 0.02]
Income		0.06	0.95	[−0.06, 0.17]
Education		0.04	0.75	[−0.07, 0.16]
Occupation		0.12	1.93	[−0.00, 0.23]
**Step 3: *MacArthur SSS scale***	0.03**			
Age		0.04	0.69	[−0.07, 0.14]
Gender		0.02	0.40	[−0.15, 0.23]
Marital status		–0.10	–1.77	[−0.45, 0.02]
Income		0.01	0.19	[−0.10, 0.13]
Education		0.04	0.68	[−0.07, 0.15]
Occupation		0.09	1.55	[−0.03, 0.21]
MacArthur SSS scale		0.19**	3.46	[0.08, 0.29]
**Step 4: *New social ladders***	0.03**			
Age		0.04	0.78	[−0.06, 0.15]
Gender		0.01	0.12	[−0.18, 0.20]
Marital status		–0.10	–1.78	[−0.44, 0.02]
Income		–0.01	–0.21	[−0.13, 0.10]
Education		–0.01	–0.21	[−0.14, 0.11]
Occupation		0.04	0.65	[−0.08, 0.16]
MacArthur SSS scale		0.07	0.97	[−0.07, 0.20]
Income ladder		0.01	0.09	[−0.13, 0.15]
Education ladder		0.14*	2.05	[0.01, 0.26]
Occupation ladder		0.18*	2.54	[0.04, 0.31]

*N* = 368; SES: Socioeconomic Status; SSS: Subjective Socioeconomic Status; Gender (1 = females, 2 = males); Marital Status (1 = involved in a romantic relationship, 2 = non-involved in a relationship). Standardized regression coefficients are reported. All VIFs ≤ 1.96. *p < 0.05, **p < 0.01, ***p < 0.001.

The standardized beta coefficient of the MacArthur SSS scale (β = 0.19, *p* = 0.001; 95% CI [0.08, 0.29]) indicated that this traditional measure of subjective SES was a positive predictor of the participants’ scores on purpose in life beyond demographics and objective SES.

Similar to the previous dimensions, our results corroborated the predictive utility of the occupation and education social ladders regarding purpose in life above and beyond demographics, indicators of objective SES, and the traditional MacArthur SSS scale. As Table 7 illustrates, participants who placed themselves higher on the occupation (β = 0.18, *p* = 0.012; 95% CI [0.04, 0.31]) and education (β = 0.14, *p* = 0.041; 95% CI [0.01, 0.26]) ladders were more prone to show greater levels of purpose in life. Moreover, the amount of explained variance of purpose in life increased by 3% in this last step of the regression analysis. The observed increase was statistically significant, *F*(3,341) = 4.17, *p* = 0.006. Also, it is worth noting that, in keeping with prior cases, the addition of the new social ladders as predictors caused the MacArthur SSS scale to no longer significantly predict purpose in life (β = 0.07, *p* = 0.333; 95% CI [−0.07, 0.20]).

#### Personal Growth

Among the various measures of objective SES (see Table 8), our results in this case yielded a significant contribution of education to the prediction of personal growth even after accounting for the respondents’ demographics (β = 0.13, *p* = 0.026; 95% CI [0.02, 0.25]).

**TABLE 8:** Hierarchical regression analysis predicting personal growth.

**Predictors**	***ΔR^2^***	**ß**	***t***	**CI (95%)**
**Step 1: *Demographics***	0.02			
Age		−0.11*	–2.10	[−0.22, -0.01]
Gender		–0.05	–0.94	[−0.29, 0.10]
Marital status		0.06	1.11	[−0.10, 0.37]
**Step 2: *Objective SES measures***	0.02*			
Age		–0.10	–1.83	[−0.21, 0.01]
Gender		–0.06	–1.09	[−0.30, 0.09]
Marital status		0.06	1.00	[−0.12, 0.36]
Income		–0.01	–0.08	[−0.12, 0.11]
Education		0.13*	2.23	[0.02, 0.25]
Occupation		–0.04	0.68	[−0.08, 0.16]
**Step 3: *MacArthur SSS scale***	0.03**			
Age		−0.11*	–2.10	[−0.22, 0.01]
Gender		–0.07	–1.29	[−0.32, 0.07]
Marital status		0.06	1.09	[−0.11, 0.37]
Income		–0.04	–0.74	[−0.16, 0.07]
Education		0.13*	2.19	[0.01, 0.24]
Occupation		0.02	0.34	[−0.10, 0.14]
MacArthur SSS scale		0.17**	3.05	[0.06, 0.28]
**Step 4: *New social ladders***	0.05**			
Age		–0.09	–1.75	[−0.20, 0.01]
Gender		–0.09	–1.67	[−0.35, 0.03]
Marital status		0.05	0.98	[−0.12, 0.35]
Income		–0.05	–0.90	[−0.17, 0.06]
Education		0.04	0.55	[−0.09, 0.17]
Occupation		–0.03	–0.46	[−0.15, 0.09]
MacArthur SSS scale		0.09	1.34	[−0.04, 0.23]
Income ladder		–0.13	–1.75	[−0.26, 0.02]
Education ladder		0.19**	2.93	[0.06, 0.32]
Occupation ladder		0.20**	2.82	[0.06, 0.33]

*N* = 368; SES: Socioeconomic Status; SSS: Subjective Socioeconomic Status; Gender (1 = females, 2 = males); Marital Status (1 = involved in a romantic relationship, 2 = non-involved in a relationship). Standardized regression coefficients are reported. All VIFs ≤ 1.96. *p < 0.05, **p < 0.01, ***p < 0.001.

As can be seen in Table 8, our results showed that the MacArthur SSS scale emerged as a significant predictor of personal growth beyond demographics and objective SES (β = 0.17, *p* = 0.002; 95% CI [0.06, 0.28]).

In line with abovementioned results concerning the new proposed ladders for income, educational level, and occupation, our results yielded a significant contribution of the occupation and education social ladders to the prediction of personal growth. As Table 8 illustrates, participants who placed themselves higher on the occupation (β = 0.20, *p* = 0.005; 95% CI [0.06, 0.33]) and education (β = 0.19, *p* = 0.004; 95% CI [0.06, 0.32]) ladders reported higher levels of personal growth even after accounting for demographics, objective SES, and the traditional MacArthur SSS scale. This last step of the regression equation explained an additional 5% of the variance in personal growth, *F*(3,341) = 5.72, *p* = 0.001. Moreover, we also observed that the standardized beta coefficient of the MacArthur SSS scale did not remain significant (β = 0.09, *p* = 0.181; 95% CI [−0.04, 0.23]) after the inclusion of the new social ladders.

#### Global Psychological Well-Being

As Table 9 illustrates, hierarchical regression analyses yielded a significant contribution of income (β = 0.14, *p* = 0.017; 95% CI [0.02, 0.25]) and education (β = 0.12, *p* = 0.036; 95% CI [0.01, 0.24]) to the prediction of the global index of psychological well-being beyond demographics.

**TABLE 9:** Hierarchical regression analysis predicting global psychological well-being.

**Predictors**	***ΔR^2^***	**ß**	***t***	**CI (95%)**
**Step 1: *Demographics***	0.00			
Age		0.03	0.59	[−0.08, 0.14]
Gender		0.05	1.00	[−0.10, 0.29]
Marital status		0.01	0.12	[−0.22, 0.25]
**Step 2: *Objective SES measures***	0.06***			
Age		0.05	0.93	[−0.06, 0.16]
Gender		0.03	0.63	[−0.13, 0.25]
Marital status		0.03	0.56	[−0.17, 0.31]
Income		0.14**	2.39	[0.02, 0.25]
Education		0.12*	2.11	[0.01, 0.24]
Occupation		0.05	0.88	[−0.07, 0.17]
**Step 3: *MacArthur SSS scale***	0.04***			
Age		0.03	0.62	[−0.07, 0.14]
Gender		0.02	0.41	[−0.15, 0.23]
Marital status		0.04	0.67	[−0.16, 0.31]
Income		0.09	1.57	[−0.02, 0.20]
Education		0.12*	2.06	[0.01, 0.23]
Occupation		0.03	0.47	[−0.09, 0.14]
MacArthur SSS scale		0.20***	3.71	[0.09, 0.31]
**Step 4: *New social ladders***	0.05***			
Age		0.05	0.88	[−0.06, 0.15]
Gender		0.00	0.09	[−0.18, 0.19]
Marital status		0.03	0.62	[−0.16, 0.30]
Income		0.07	1.18	[−0.05, 0.18]
Education		0.03	0.52	[−0.09, 0.16]
Occupation		–0.03	–0.42	[−0.15, 0.09]
MacArthur SSS scale		0.07	1.00	[−0.07, 0.20]
Income ladder		–0.01	–0.15	[−0.15, 0.13]
Education ladder		0.20**	3.09	[0.07, 0.33]
Occupation ladder		0.18**	2.61	[0.04, 0.31]

*N* = 368; SES: Socioeconomic Status; SSS: Subjective Socioeconomic Status; Gender (1 = females, 2 = males); Marital Status (1 = involved in a romantic relationship, 2 = non-involved in a relationship). Standardized regression coefficients are reported. All VIFs ≤ 1.96. *p < 0.05, **p < 0.01, ***p < 0.001.

We also found that the MacArthur SSS scale predicted global psychological well-being. In particular, it was a positive predictor of this global index (β = 0.20, *p* < 0.001; 95% CI [0.09, 0.31]) beyond demographics and objective SES.

As occurred in earlier cases, standardized beta coefficients indicated that the education (β = 0.20, *p* = 0.002; 95% CI [0.07, 0.33]) and occupation (β = 0.18, *p* = 0.010; 95% CI [0.04, 0.31]) ladders were positive predictors of global psychological well-being even after accounting for demographics, objective SES, and the MacArthur SSS scale (see Table 9). The amount of explained variance of global psychological well-being increased by 5% in this last step of the regression analysis. The observed increase was statistically significant, *F*(3,341) = 6.11, *p* < 0.001. In addition, it is worth noticing that the addition of the new social ladders as predictors caused the MacArthur SSS scale to no longer significantly predict global psychological well-being (β = 0.07, *p* = 0.319; 95% CI [−0.07, 0.20]).

**FIGURE 2:**
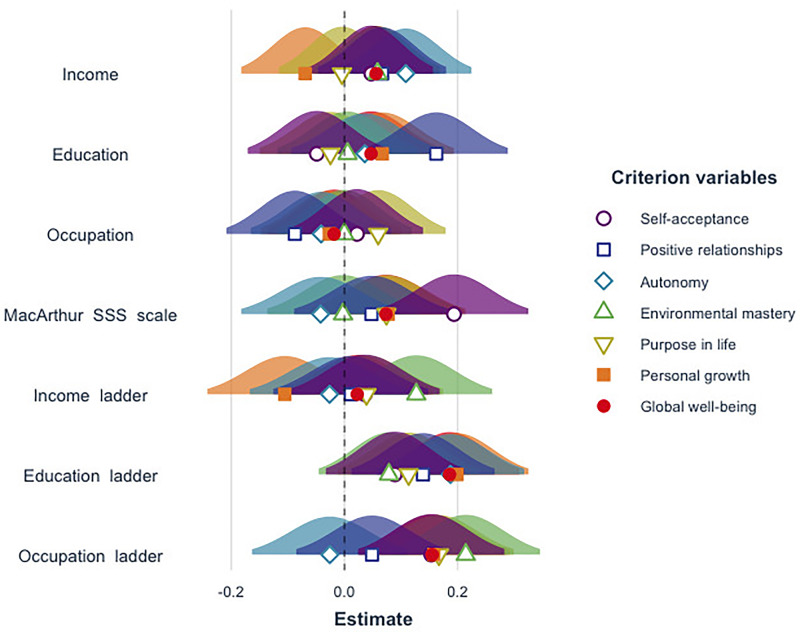
Visual comparison of standardized beta coefficients of the various objective and subjective SES measures.

Lastly, we graphically illustrate the standardized beta coefficients of the different measures of objective and subjective SES in Figure 2.

## Discussion

Prior research has widely converged on the notion that SES is a rather complex and multifaceted construct determined by relatively independent objective indicators (e.g., income, educational level) and individuals’ subjective perceptions of their placement in the socioeconomic hierarchy (see Kraus et al., 2012). Nonetheless, within the psychological literature on subjective SES and well-being, subjective assessments of SES have largely focused on the administration of social ladders that simultaneously consider income, educational level, and occupation, potentially constraining individuals’ views of their SES. In an attempt to address this gap, the present research revisited subjective SES measurement by (a) proposing a novel method of assessing subjective SES, namely an adaption of the MacArthur SSS scale, resulting in three independent ladders based on income, educational level, and occupation, and (b) empirically testing the role of these three subjective SES measures in psychological well-being while examining in conjunction objective SES and the traditional MacArthur SSS scale. Hence, this investigation provides the first preliminary data on the empirical contribution of distinctive social ladders focused on income, education, and occupation, as an innovative and broader way of evaluating the effects of subjective SES on various components of psychological well-being.

In aligning with notions recognized in earlier studies, our results clearly confirmed that subjective assessments of SES are better predictors of well-being-related aspects than are objective SES metrics (e.g., Boyce et al., 2010; Cundiff and Matthews, 2017; Navarro-Carrillo et al., 2019). Indeed, among the various components of psychological well-being (i.e., self-acceptance, positive relationships, autonomy, environmental mastery, purpose in life, and personal growth), only the participants’ differences in positive relationships and autonomy with others were significantly explained by an objective SES index while all SES indicators were simultaneously considered. In particular, higher educational level predicted greater scores on positive relationships, and higher income predicted increased autonomy. However, it is important to mention that the education ladder, compared to income, exhibited a higher predictive utility regarding autonomy.

In considering the role of subjective SES indicators, we found that participants’ higher placements on the traditional MacArthur SSS scale only significantly predicted greater scores on self-acceptance when we simultaneously considered the predictive utility of the new proposed ladders for income, educational level, and occupation. Moreover, the traditional MacArthur SSS scale was not a significant predictor of global psychological well-being. In particular, our findings indicated that the novel indicators of subjective SES were stronger predictors of all measures of psychological well-being except self-acceptance than the conventional MacArthur SSS scale. In addition, their inclusion in the hierarchical regression analyses significantly accounted for incremental criterion variance (except in positive relationships) beyond demographics, objective SES, and the MacArthur SSS scale. Interestingly, of these new social ladders, the one linked to income levels, did not emerge as a significant predictor of any of the criterion indicators. Taking into account that income, when compared to other objective facets of SES (e.g., education), has been found to exhibit stronger associations with well-being (e.g., Tan et al., in press), one might argue that the income ladder should show similar effects. However, according to our data, the education and occupation ladders were identified as consistent predictors of psychological well-being. The education ladder was significantly related to all indicators of psychological well-being except self-acceptance and environmental mastery, which were explained better by the occupation ladder. These results concerning the education ladder could help to elucidate the role of this facet of SES in psychological well-being. Although the contribution of education to well-being has been shown to be relatively limited when it is objectively assessed (Tan et al., in press), our findings highlight its relative relevance to psychological well-being when individuals estimate their position within the social hierarchy by comparing their educational level to that of others in society. Previous works have posited that education may precede higher income levels or more prestigious occupations (Snibbe and Markus, 2005). Furthermore, the role of the occupation ladder was almost comparable to that of the education ladder. In particular, we found the occupation ladder predicted all measures of psychological well-being except positive relationships and autonomy, which were explained better by the education ladder. In this case, a similar interpretation to that proposed for the education ladder might be extrapolated for the occupation last. However, the characteristics of the socioeconomic context should not be overlooked. Specifically, this research was conducted in southeastern Spain. This region’s overall socioeconomic reality, even some time after the economic crisis period, remains unfavorable. Thus, within this context of economic difficulties and high unemployment, the perceived value of having a more prestigious occupation might be crucial for individuals’ psychological well-being. Together, our findings revealed that the novel education and occupation ladders are unique predictors of psychological well-being beyond objective SES, and the traditional MacArthur SSS scale.

### Limitations and Future Research Directions

Although the current findings allow open up a new strand of research in the psychological literature on SES and well-being, some limitations should be acknowledged while suggesting further research directions. First, it is worth mentioning that this study used non-probabilistic sampling (i.e., snowball sampling procedure), which constrains our results’ potential generalization. It should also be noted here that, although the present sample consisted of well-educated individuals of relatively higher occupational status, respondents’ characteristics in terms of income generally were equivalent to the reference population (INE, 2019). Overall, future research should use probabilistic sampling procedures to collect samples that are as representative as possible. In addition, we followed a non-experimental methodology in this research. Hence, causal inferences regarding our findings must not be made. Thus, further research should use experimental or longitudinal designs to determine the potential causal effects of the proposed subjective SES measures on psychological well-being. Second, although we included different well-established dimensions of psychological well-being (Ryff and Keyes, 1995; Díaz et al., 2006) as relevant criteria, it would be advisable to evaluate further well-being or health-related factors (e.g., self-perceived health status or physical health). By doing so, future researchers could rule out the possibility that our results are due in some way to the type of criteria included, thereby extending and complementing this study. Third, our research was carried out in a specific sociocultural context (Spain). Although suggestive, and consistent with the notion that subjective assessments of SES are better predictors of well-being than objective SES indicators, our findings do not allow us to articulate a comprehensive explanation of why subjective SES outperform objective SES in explaining people’s well-being differences. Nonetheless, these results could be useful for subsequent empirical studies aimed at unraveling this issue. Lastly, the present findings should be expanded with new investigations to test these connections in other countries, as well as potential moderators from inequality levels (Cheung and Lucas, 2016) to cultural dimensions (Curhan et al., 2014).

## Conclusion

This research offers valuable preliminary insight into the fields of SES and well-being and health by presenting a new approach for subjective SES estimation and testing the empirical contribution of this innovative measurement strategy to psychological well-being. In particular, our findings confirmed that the novel education and occupation ladders are predictive of a significant proportion of the variance levels of psychological well-being that are not due to objective SES metrics (i.e., income, education, and occupation) or the conventional MacArthur SSS scale, underlining the need to validate and expand the present results across samples for the sake of a better understanding of the SES–well-being/health connection.

## Data Availability Statement

The datasets generated for this study are available on request to the corresponding author.

## Ethics Statement

This research study was approved by the ethical committee of the southeast Spanish university and carried out in compliance with the Ethical Standards of the 1964 Declaration of Helsinki.

## Author Contributions

GN-C, MA-F, and IV-S collected the data of the study. GN-C, MA-F, MM, and IV-S analyzed and interpreted the data and reviewed and edited the manuscript. GN-C and MA-F wrote the original draft.

## Conflict of Interest

The authors declare that the research was conducted in the absence of any commercial or financial relationships that could be construed as a potential conflict of interest.
